# Supplementary figures and images for: Modulations of microbehaviour by associative memory strength in Drosophila larvae
Source: PLoS One. 2019 Oct 21;14(10):e0224154. doi: 10.1371/journal.pone.0224154 (PMC6802848; doi:10.1371/journal.pone.0224154)

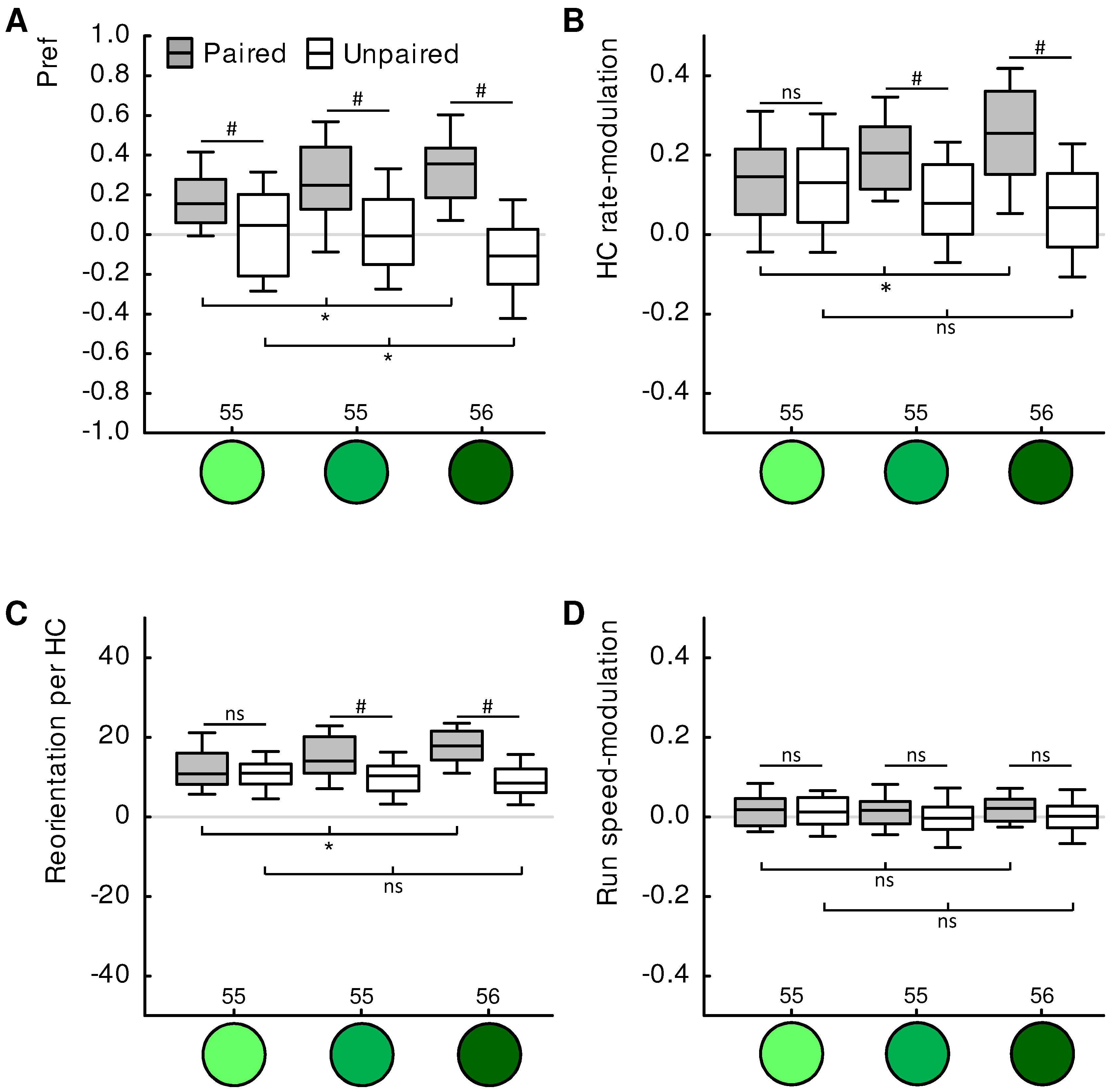

Supplement: S1 Fig — Displayed are the results for (A) Pref, (B) HC rate-modulation, (C) Reorientation per HC and (D) Run speed-modulation underlying the data shown in Fig 1B–1E. Results after paired training are displayed in grey-filled box plots, whereas results after unpaired training are displayed in white box plots. Asterisks indicate significant Kruskal-Wallis tests (KW) across all the paired-trained or all the unpaired-trained groups, respectively (p < 0.05 corrected according to Bonferroni-Holm); hash signs indicate significant Mann-Whitney U-tests (MW) between paired-trained and unpaired-trained groups (p < 0.05 corrected according to Bonferroni-Holm). Odour preference, HC rate-modulation and Reorientation per HC, but not Run speed-modulation, were significantly affected by the FRU concentration during paired training (KW [A] H = 15.7, df = 2, p = 0.0004; [B] H = 18.2, df = 2, p = 0.0001; [C] H = 21.1, df = 2, p < 0.0001; [D] H = 0.8, df = 2, p = 0.68). As regards unpaired training, only odour preference was significantly affected by the FRU concentration, whereas HC rate-modulation, Reorientation per HC and Run speed-modulation were not (KW [A] H = 10.3, df = 2, p = 0.0057; [B] H = 5.9, df = 2, p = 0.053; [C] H = 4.3, df = 2, p = 0.11; [D] H = 2.6, df = 2, p = 0.28). At the lowest FRU concentration, odour preference differed significantly after paired and unpaired training, but none of the aspects of chemotaxis did (MW, [A] U = 1008, p = 0.0011; [B] U = 1475, p = 0.59; [C] U = 1458, p = 0.53; [D] U = 1499, p = 0.70). Higher concentrations and therefore stronger memories correspond to significant differences after paired and unpaired training in odour preference, HC rate-modulation and Reorientation per HC, but not Run speed-modulation (MW [A] U = 721, p = 0.00001; U = 258, p < 0.00001; [B] U = 690, p < 0.00001; U = 521, p < 0.00001; [C] U = 792, p < 0.00001; U = 432, p < 0.00001; [D] U = 1281, p = 0.10; U = 1198, p = 0.032). Sample sizes are indicated below each box plot. Box [file pone.0224154.s001.tif]

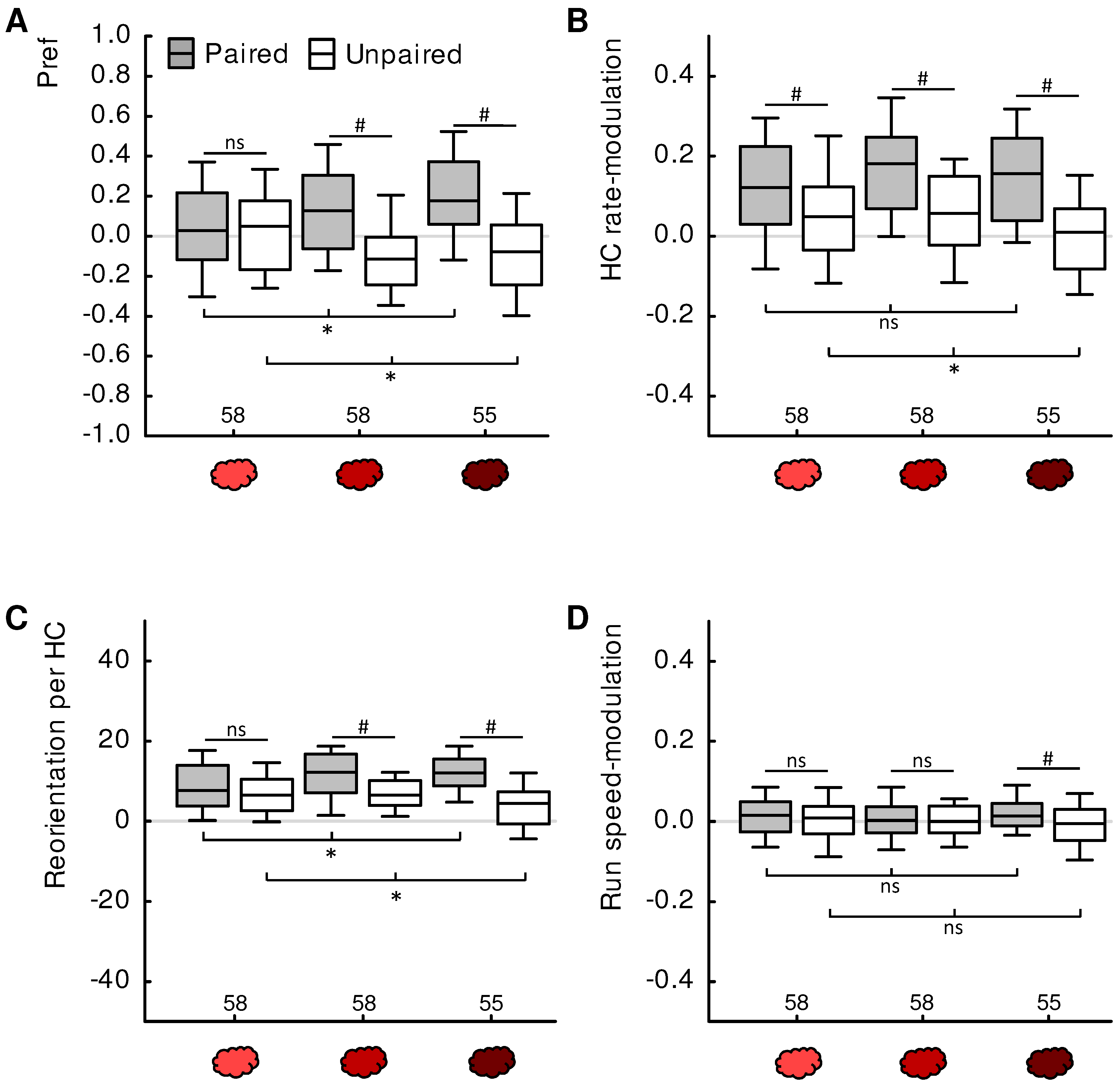

Supplement: S2 Fig — Displayed are the results for (A) Pref, (B) HC rate-modulation, (C) Reorientation per HC and (D) Run speed-modulation underlying the data shown in Fig 1G–1J. Results after paired training are displayed in grey-filled box plots, whereas results after unpaired training are displayed in white box plots. Odour preference and Reorientation per HC, but not HC rate-modulation or Run speed-modulation, were significantly affected by the AM concentration during paired training (KW [A] H = 9.9, df = 2, p = 0.0071; [B] H = 3.2, df = 2, p = 0.20; [C] H = 9.1, df = 2, p = 0.011; [D] H = 2.8, df = 2, p = 0.25). As regards unpaired training, odour preference, HC rate-modulation and Reorientation per HC were significantly affected by the AM concentration, whereas Run speed-modulation was not (KW [A] H = 10.0, df = 2, p = 0.0066; [B] H = 7.9, df = 2, p = 0.02; [C] H = 10.8, df = 2, p = 0.0046; [D] H = 1.3, df = 2, p = 0.52). At the lowest AM concentration, only HC rate-modulation differed after paired and unpaired training (MW, [A] U = 1610, p = 0.69; [B] U = 1216, p = 0.010; [C] U = 1401, p = 0.12; [D] U = 1561, p = 0.66). At the medium AM concentration, odour preference, HC rate-modulation and Reorientation per HC, but not Run speed-modulation, were different after paired and unpaired training (MW, [A] U = 915, p = 0.00002; [B] U = 915, p = 0.00002; [C] U = 885, p = 0.00001; [D] U = 1672, p = 0.95). At the highest AM concentration, all scores were significantly different after paired and unpaired training (MW, [A] U = 611, p < 0.00001; [B] U = 643, p < 0.00001; [C] U = 489, p < 0.00001; [D] U = 1162, p = 0.013). Sample sizes are indicated below each box plot. For further details, see S1 Fig. (TIF) [file pone.0224154.s002.tif]

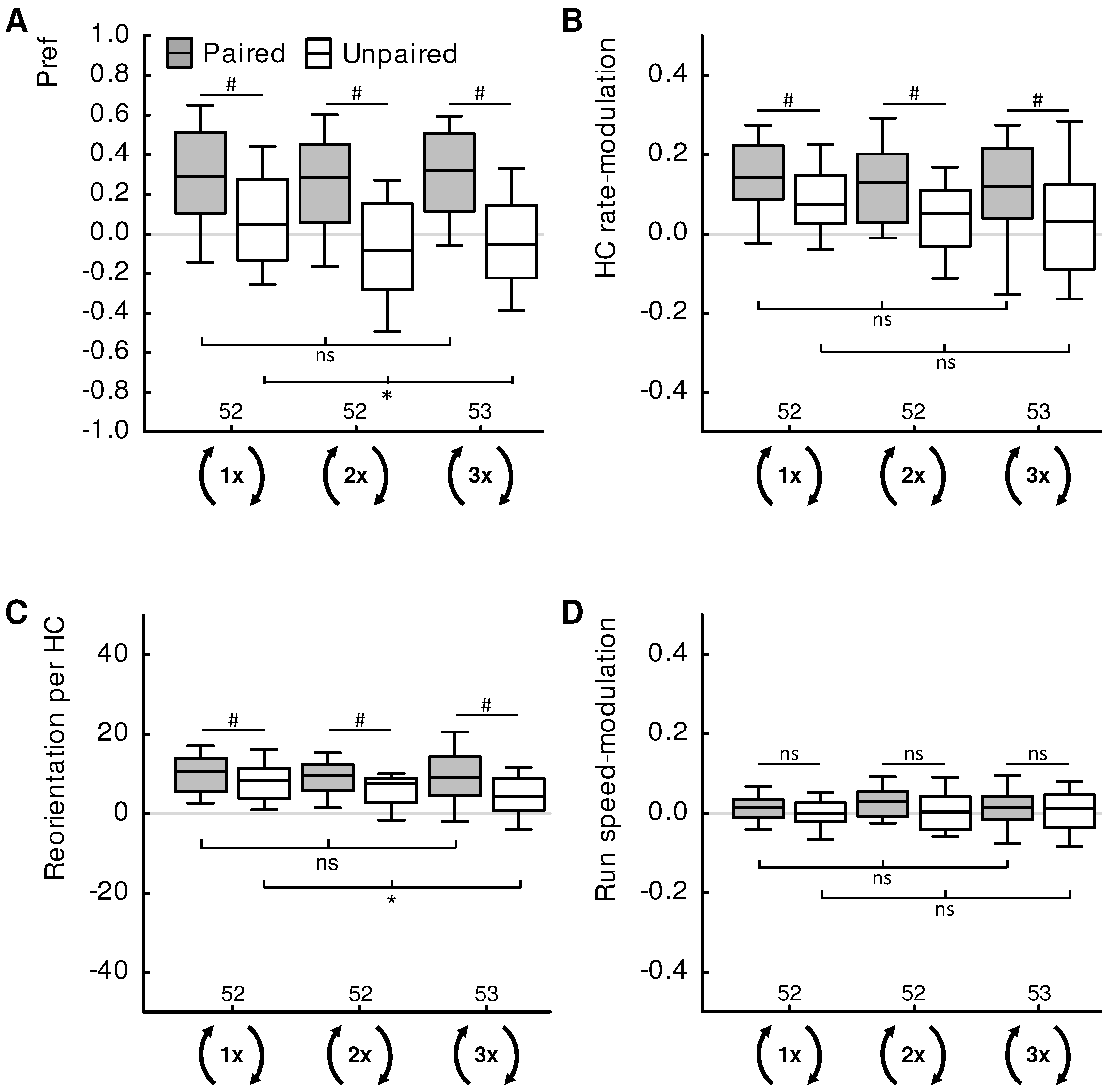

Supplement: S3 Fig — Displayed are the results for (A) Pref, (B) HC rate-modulation, (C) Reorientation per HC and (D) Run speed-modulation underlying the data shown in Fig 1L–1O. Results after paired training are displayed in grey-filled box plots, whereas results after unpaired training are displayed in white box plots. None of the measured aspects of chemotaxis was significantly changed by varying the number of paired-training trials (KW [A] H = 1.1, df = 2, p = 0.58; [B] H = 0.9, df = 2, p = 0.64; [C] H = 1.0, df = 2, p = 0.61; [D] H = 2.7, df = 2, p = 0.26). Odour preference and Reorientation per HC were significantly affected by the number of unpaired-training trials, whereas HC rate-modulation and Run speed-modulation were not (KW [A] H = 7.5, df = 2, p = 0.023; [B] H = 3.8, df = 2, p = 0.15; [C] H = 8.8, df = 2, p = 0.012; [D] H = 0.8, df = 2, p = 0.67). Irrespective of the number of training trials, odour preference, HC rate-modulation and Reorientation per HC differed after paired and unpaired training, whereas Run speed-modulation did not (MW from left to right: [A] U = 858, p = 0.00057; U = 613, p < 0.00001; U = 545, p < 0.00001; [B] U = 900, p = 0.0015; U = 830, p = 0.00045; U = 995, p = 0.0067; [C] U = 1084, p = 0.043; U = 923, p = 0.0036; U = 909, p = 0.0012; [D] U = 1153, p = 0.11; U = 1043, p = 0.032; U = 1350, p = 0.62). Sample sizes are indicated below each box plot. For further details, see S1 Fig. (TIF) [file pone.0224154.s003.tif]
